# The effectiveness of a model-based health education program on genital warts preventive behaviors: a quasi-experimental study

**DOI:** 10.1186/s13027-021-00408-w

**Published:** 2021-12-11

**Authors:** Zahra Hosseini, Niloofar Seyrafi, Teamur Aghamolaei, Shokrollah Mohseni, Azin Alavi, Sakineh Dadipoor

**Affiliations:** 1 Social Determinants in Health Promotion Research Center, Hormozgan Health Institute, Hormozgan University of Medical Sciences, Bandar Abbas, Iran; 2 Student Research Committee, Hormozgan University of Medical Sciences, Bandar Abbas, Iran; 3 Cardiovascular Research Center, Hormozgan University of Medical Sciences, Bandar Abbas, Iran; 4 Mother and Child Welfare Research Center, Faculty of Nursing and Midwifery, Hormozgan University of Medical Sciences, Bandar Abbas, Iran; 5 Infectious and Tropical Diseases Research Center, Hormozgan Health Institute, Hormozgan University of Medical Sciences, Bandar Abbas, Iran

**Keywords:** Genital warts, Human papillomavirus, Women, Health belief model

## Abstract

**Background:**

Genital warts (GWs) are highly prevalent among Iranian women. GWs are not only highly infectious but are also followed by severe adverse effects, including the development of cervical cancer. Therefore, the present study aimed to explore the effect of an educational intervention based on the health belief model (HBM) on the adoption of GWs preventive behaviors by married women in Bandar Abbas, a city in the south of Iran.

**Methods:**

A quasi-experimental intervention was conducted between 2019 and 2020 among 150 women participants (75 as the intervention and 75 as the control group). The sampling method was multi-stage clustering. The required data was collected using a reliable and valid tripartite questionnaire which explored demographic information, awareness, and HBM constructs. A total number of 15 educational sessions were held, each 90 min long. The control group received only one 90-min session. The final follow-up was completed three months after the intervention in November 2020.

**Results:**

The two research groups had no statistically significant differences in terms of awareness, perceived susceptibility, severity, benefits, barriers, and self-efficacy before the intervention (in the pre-test) (*p* > .05). After the educational intervention, the two groups showed statistically significant differences in all constructs except for the perceived benefits (*p* < .001). In the intervention group, in the pretest (before the intervention), the behavior score was 2.77 ± 2.59, which was increased to 3.73 ± .52 after the intervention (*p* < .001). In the control group, however, the difference was not statistically significant (*p* = 0.227).

**Conclusion:**

The present findings showed that the educational intervention based on the HBM can improve the prevalence of GWs preventive behaviors in women. This education should be provided by experts at regular intervals in all healthcare centers.

**Supplementary Information:**

The online version contains supplementary material available at 10.1186/s13027-021-00408-w.

## Background

HPV is a prevalent infection that is transmitted through sexual contact. It is related to cancers such as cervical, head and neck squamous cell cancer and anal cancer [1]. HPV genotypes exceed 120 types. More than 40 of them affect the anogenital epithelium of men and women. About 14 HPV genotypes are called high risk as they can lead to cervical cancer and account for a great many cancers in the vagina, vulva, penis, anus, or oropharynx [2]. A number of low-risk HPV genotypes can lead to GWS (condyloma acuminate) [3]. About 70% of cervical cancers are induced by HPV 16 and 18. Moreover, 90% of GWS are induced by genotypes 6 and 11 [4]. Several studies found a statistically significant correlation between GWs and some cancers in the reproductive system [5, 6]. A study reported that the incidence rate of cancers associated with genitals is higher in women afflicted with GWs [7].

GWs are among the most prevalent viral and infectious sexually transmitted diseases. About 27% of sexually active people suffer from the disease. A body of research shows that in 60% of cases, a single sexual contact via skin and oral mucosa can transmit the disease to the sex partner [8]. The related literature show that about 6.2 million new types of GWs occur annually in the 14–44-year-old population [9]. The findings on the prevalence of GWs in different countries have been quite divergent. One study reported the incidence rate of GWs among Indian women to be between 1.4 and 25% [10]. Another study reported the prevalence of GWs in Italian and Philippine women to be 0.038% and 3.39%, respectively [11]. The prevalence of GWs is high among Iranian women [12–14]. A study in Iran showed that 20.8% of women who had been referred to the clinic for vaginal infection were diagnosed with GWs [13]. Another study showed that 47.3% of GWs occurred in the 24–30 year age group [15]. In their research on 851 Iranian women, Shafaghi et al. found that 265 subjects were afflicted with different types of HPV and some with GWs [16].

GWS are highly infectious, and about 65% of people who have infected sex partners get afflicted with the disease in 3–8 months [17]. GWs are not only highly contagious but also accompanied by clinical symptoms such as an itchy or pricking feel, pain, hemorrhage and the development of cervical cancer [18]. An early diagnosis might need no special treatment. Yet, diagnosis with GWs can have significant social and psychological effects on patients. A body of research showed that GWs can change the appearance of the vulva, causing embarrassment and anxiety and the quality of sexual relationships [10]. As there is no HPV vaccination yet in Iran, and sexual relationships are only acceptable for married women in the culture, the sexually transmitted infection test is quite a challenge for most women [19]. It seems that preventing infection is the best strategy in this region. Another study reported that preventing GWs-related high-risk behaviors can be effective in controlling the disease [18]. Moreover, preventing risky behaviors can largely contribute to women's awareness, attitude, and beliefs [20].

Several studies in Iran reported a low level of young women and girls’ awareness of GWs and HPV. These studies also indicated that women are not educated properly and have limited knowledge of the hazards of the virus and its role in the occurrence of cervical cancer [13, 21, 22]. In fact, only 8% of people afflicted with GWs use condoms [23]. However, in Hong Kong, 65% of the population afflicted with HPV use condoms [24]. These facts and figures reveal the lack of GWs preventive behaviors in Iran. Another study showed that raising awareness of GWs can significantly help to reduce its epidemic [8]. Therefore, the first step to control the disease is to raise awareness of the infection and adopt the required preventive and protective behaviors. Presumably, awareness-raising affects people’s attitude, susceptibility and perceived risks and, thus, plays a preventive role in affliction with GWs.

Intervention design is informed by health behaviours models and theories. The Health Belief Model (HBM) is one such model and has been shown to be effective in driving change in sexual behaviours [25–27]. If people perceive themselves as susceptible to the disease and perceive the benefits of staying away from infection, they are motivated to change their behaviors. The HBM encompasses the perceived benefits and barriers within its expected value framework. If the perceived benefits are more than perceived barriers, there are higher chances of adopting a healthy behavior [28, 29].

This model illuminates how one’s perception can create a certain motivation or behavior. According to this model, in order to adopt the desired preventive behavior, people should first perceive themselves as susceptible to GWs, and then perceive the severity of the disease and the different physical, social, psychological and economic side effects through the cues to action from the surrounding environment or the inner world. They need to believe in the practicality of the GWs preventive program and perceive the benefits of the program and find the benefits of the behaviors more than the barriers. They should see the program as less costly and see themselves capable of showing preventive behaviors. This is undertaken as self-efficacy within the theoretical model. Taking all these into account, people are ready to show GWs preventive behaviors [29].

A body of research reported significant correlations between GWs and vulva, vagina, penis, anus, anogenital malignancies, head and neck cancers [5, 6, 30]. Considering the correlation between GWs and malignancies in genitals, the present research aimed to investigate the effect of an educational intervention based on the health belief model on the adoption of GWs preventive behaviors in women visiting healthcare centers in Bandar Abbas.

## Methods

### Design and sampling

The present quasi-experimental research with an experimental and a control group was conducted in 2019–2020.

It aimed to explore the effect of an educational intervention based on the HBM on the adoption of GWs preventive behaviors through a 3-month follow-up among married women above 15 years old in Bandar Abbas, Iran.

### Setting

The present research was carried out in Bandar Abbas, the capital city of Hormozgan Province in the south of Iran. This city is located in 27.19 latitudes and 56.28 longitudes and is located 9 m above sea level. Bandar Abbas has a population of 352,173, which makes it the largest city in Hormozgan Province.

### Eligibility criteria

The inclusion criteria were an age above 15 years, no history of sexually transmitted diseases, being married and being local to Bandar Abbas (born in the city or living there for at least ten years for cultural adjustment).

Signing an informed consent to take part was another inclusion criterion. The exclusion criteria were absence for more than two sessions, unavailability in the post-test, and the omission of incomplete questionnaires.

### Intervention and follow-up

The questionnaires were (knowledge and HBM constructs) were submitted to participants in both the intervention and control groups before the experiment. The pretest results were used for a needs analysis of educational content, teaching methods and the number of sessions needed for education. The educational sessions were held for the intervention group in a friendly environment such as a local mosque or a healthcare center within a four month’s period. The material to be taught was developed based on the learners' comprehension, reliance on valid sources, professors' comments and participants' views based on the HBM. In total, 15 educational sessions were held (14 held by the first author and one by a gynecologist). There were six instructional groups. Each session was 40–60 min in length with a 10-min interval. The teaching techniques used included lectures, cooperative discussions, brainstorming, photos and movies. The following content was covered in the educational intervention: (1) familiarity with HPV infection, all symptoms of GWs, ways of transmitting GWs. (2) GWs risk factors (3) the significance of preventing the incidence of GWs (4) the short-term and long-term adverse effects of GWs (5) benefits of adopting GWs preventive behaviors (6) using useful strategies to remove barriers to adopting healthy behavior (7) motivating women to adopt GWS preventive behaviors and increasing self-efficacy. The details on the educational sessions are indicated in Additional file 1. Of note is that to our knowledge, Iranian women are less familiar with the term HPV. They mainly know HPV as only associated with GWs. Thus, the resent researchers tried to use GWs more, but developed the educational content of sessions so as to raise awareness of HPV in general. The control group received 1 educational session on how GWs are transmitted and the significance of personal healthcare in preventing the spread of the disease. Three months after the intervention, the posttest questionnaires were filled out in both research groups to assess the effectiveness of the intervention**.**

### Sample size estimation

The present research aimed to compare the scores for HBM constructs between the control and intervention groups. Thus, a formula was used to estimate the sample size needed for acquiring information from two independent groups, as indicated below. In similar research entitled as "Investigating the effect of an educational intervention based on the HBM on administering the Pap test among women in Fasa," Khiali et al. [31]. Reported the mean and standard deviation of perceived severity in the intervention and control groups 20.86 ± 2.13 and 18.95 ± 2.99, respectively. The following formula was used with α = 0.05 ($z_{{1 - \frac{\alpha }{2}}} \simeq 2$), β = 0.2 ($z_{1 - \beta } = 0.85$). The least difference of means (the effect size) was 1. The eventual sample size for each group was estimated at 73 in the formula. To be on the safe side, a total number of 75 participants was set for each research group.

$$n = \frac{{(z_{{1 - \frac{\alpha }{2}}} + z_{1 - \beta } )^{2} (\delta_{1}^{2} + \delta_{2}^{2} )}}{{(\mu_{1} - \mu_{2} )^{2} }} = \frac{{(2 + 0.85)^{2} (2.13 + 2.99)^{2} }}{1} = 73 \Rightarrow \, \approx 75$$

### Sampling type

In Iran, the sampling was done as multi-stage. In other words, in the first stage, the overall 20 comprehensive healthcare centers in Bandar Abbas were divided into five geographical regions (north, south, east, west, and center). Each geographical region included four comprehensive healthcare centers. In the second stage, two healthcare centers were selected randomly from each region (in total, ten comprehensive healthcare centers). In the third stage, five centers were randomly selected (1 center from each geographical region) and assigned to the intervention group and 5 to the control group. Finally, in each center, the sample was selected from the visitors to healthcare centers. If they met the inclusion criteria, they were selected through convenience sampling method until the required sample size (n = 15) was met.

### Data collection

The pretest questionnaires were provided to subjects in both the control and intervention groups who had provided written consent to take part in the study. At the end of the 15th education session, both the control group and the intervention group completed the post-intervention questionnaire. To facilitate the identification of the pre- and post-test questionnaires, the last four digits of each participant's mobile phone number and age were coded on the questionnaires. Each questionnaire took 20–25 min to complete. The researcher was not present during the questionnaire completion process. For illiterate participants, the content of the questionnaire was read out loud and answered with the least possible bias with the help of a member of the research team.

### Instrumentation and scoring system

The questionnaire used in this research contained closed-ended questions to be answered on a Likert type: True/False/Don’t Know. There were three parts to the questionnaire as introduced below.

### Part 1: Demographic information

Participants' demographic information included their age described at two levels (≤ 30 and ≥ 31), education (below diploma, diploma, higher), husband's education (below diploma, diploma, higher), occupation (not working, working outside the home).

### Part 2: Awareness level

There were 24 items about awareness of GWs. The reliability of this part of the questionnaire was tested by Farshbaf et al. among women in Tabriz City and was found to be 82% [32]. Items enquiring about the awareness of GWs were rated on a three-point Likert type (True, False, Don’t Know). ‏ A correct answer was scored as 1 and an incorrect answer was scored as zero. These items explored certain aspects of awareness, including how the disease can be transmitted, the symptoms of the disease and how to prevent GWs.

### Part 3: HBM constructs

There were 6 sub-scales in this part of the questionnaire. “Perceived susceptibility” included 7 items an instance of which is “Despite taking genital care into account, I am still susceptible to genital warts”. “Perceived severity” was tested with 5 items one of which is: “Affliction with GWs may make me infertile." "Perceived benefits" was rated on 7 items and explored the extent to which the participants perceived the benefits of an early diagnosis of GWs to prevent severe symptoms. An instance of the content is: “It did not cost me to adhere to health recommendations."

The “Perceived barriers” to the adoption of GWs preventive behaviors were tested along with ten items including "I feel embarrassed if others know I am afflicted with GWs." "Self-efficacy" was tested with six items to explore the participants' capability of showing GWs preventive behaviors. An instance is: "I am sure I can tolerate the little pain of the Pap test to take care of myself". Finally, the healthy behavior was tested with four items, including: "I follow genital healthcare advice to lower the chances of affliction with GWs."

The above-mentioned items were all tested on a 5-point Likert type: (1) strongly agree, (2) agree, (3) neutral, (4) disagree, (5) strongly disagree. At the end, the scores were not added up. Rather, they were calculated separately and reported for each participant. A higher score showed stronger feelings towards the construct. All subscales were positively related to GWs preventive behavior except for perceived barriers which was negatively correlated.

### Data quality assurance

Pretested and structured questions were used as the data collection instrument after a review of the most recent related papers. All questionnaire items were answered as self-reports. These were pretested on 23 women with similar characteristics to the target population. Their comments were used to revise the questionnaire and better organize it. These participants were excluded from the main data collection phase. The first draft of the questionnaire was also checked by a panel of experts for readability, simplicity, relatedness and importance of content. Their comments were used to further revise the draft. The test–retest method was used to check the reliability. To this aim, the questionnaire was submitted at a two-week interval to 20 respondents who met similar conditions to the main participants. Then, the ICC index was used to check the consistency of scores from the first administration to the next. The estimated ICC was 0.83.

### Ethical considerations

A formal permission letter was obtained for data collection from the university deputy of research. Then, the target healthcare centers were visited. Upon admission, the researcher introduced herself completely and elaborated on the purpose of research for the participating women. Then a written letter of consent was obtained from all participants with all details of the research procedure. Participation was voluntary. All participants were asked not to mention their name and were all assured of the confidentiality of the information they provided. This research was approved by the committee of ethics at Hormozgan University of medical sciences.

### Data management and analysis

To describe quantitative variables (i.e., age, HBM constructs), mean and standard deviation were used. To describe qualitative variables (i.e., age group, education, husband's education and occupation), frequency and relative frequency were used. To test the parametric hypotheses such as the normality of distribution and equal variance, Kolmogorov–Smirnov and Levene’s test were used. Independent samples T-test was used then to compare the HBM constructs between the control and intervention groups as well as the adoption of GWs preventive behaviors between the two groups. Paired-samples T-test was run to compare changes in the scores of the model constructs in each group in the pre-and post-test. ANCOVA was run to moderate and control the effect of the pretest scores on the posttest. Multiple linear regression analysis was used in the intervention group to test the effect of each model construct on the preventive behavior score. Behavior was used as the dependent variable and awareness and model constructs as the independent variables. All analyses were done in SPSS20.

## Results

### Descriptive phase: socio-demographic characteristics

The present quasi-experimental research was conducted on 150 women (75 in the intervention and 75 in the control group) between 17 and 42 years of age. The mean and standard deviation of the participants’ age was 26.09 ± 4.15 and 28.35 ± 6.6 in the control and intervention groups, respectively. Concerning education, in both groups, the highest frequency was that of diploma. In the control and intervention groups, it was 38 (50.7%) and 29 (38.7%), respectively. As for occupation, in both groups, the highest frequency belonged to the housewives. 56 participants (74.4%) of the control group and 51 of the intervention (68%) were housewives. The other demographic variables are described in Table 1.

**Table 1:** Participants’ demographic information in the research groups

Variables	Total sample N (150)	Intervention group (n = 75)	Control group (n = 75)	*p* value
*Age (years)*				
≤ 30	114 (76%)	54 (72%)	60 (80%)	0.014
≥ 31	36 (24%)	21 (28%)	15 (20%)	
*Educational level*				
Below diploma	31 (20.66%)	20 (26.7%)	11 (14.7%)	0.148
Diploma	67 (44.66%)	29 (38.7%)	38 (50.7%)	
Higher	52 (34.66%)	26 (34.7%)	26 (34.7%)	
*Husband’s education*				
Below diploma	33 (22%)	17 (22.7%)	16 (21.3%)	0.977
Diploma	52 (34.66%)	26 (34.7%)	26 (34.7%)	
Higher	65 (43.33%)	32 (42.7%)	33 (44%)	
*Occupation*				
Not working (housewife)	107 (71.34%)	51 (68%)	56 (74.4%)	0.726
Working outside home	43 (28.66%)	24 (32%)	19 (25.3%)	

### Awareness and HBM scores in the pre- versus post-test

In the pretest, the between-group difference was not statistically significant in terms of awareness and the HBM constructs (*p* > 0.05). However, in the posttest, the between-group difference was statistically significant in terms of the awareness score and HBM constructs, but not for the perceived benefits (*p* < 0.001). In the pretest, the behavior score in the intervention was 2.77 ± 2.59, which was increased to 3.73 ± 0.52 in the post-test. This difference was statistically significant. In the control group, the difference in the behavior score was not statistically significant in the pre- and posttest (Table 2).

**Table 2:** Comparison of HBM constructs in the pre- and post-test in two research groups

Variables	Groups	Pre-test (Mean ± SD)	Post-test (Mean ± SD)	*p* value
Awareness	Intervention	14.21 ± 5.26	18.68 ± 1.66	< 0.001
	Control	14.49 ± 5.56	15.55 ± 1.36	0.115
	*p* value	0.752	< 0.001	
Perceived susceptibility	Intervention	18.71 ± 5.52	26.27 ± 3.12	< .001
	Control	18.52 ± 6.4	18.77 ± 3.30	.717
	*p* value	.844	< .001	
Perceived severity	Intervention	18.40 ± 4.78	23.84 ± 3.05	< .001
	Control	18.57 ± 4.04	19.16 ± 3.09	.284
	*p* value	.812	< .001	
Perceived barriers	Intervention	29.29. ± 8.57	25.88. ± 5.27	< .001
	Control	29.83 ± 7.62	28.89 ± 4.52	.0770
	*p* value	.687	< .001	
Perceived benefits	Intervention	23.99 ± 2.23	24.32 ± 0.47	.314
	Control	23.79 ± 3.43	23.96 ± 3.72	.716
	*p* value	.693	.343	
Self-efficacy	Intervention	20.03 ± 4.04	23.12 ± 1.95	< .001
	Control	20.04 ± 5.75	20.39 ± 2.29	.543
	*p* value	.987	.001	
Behavior	Intervention	2.77 ± 2.59	3.73 ± 0.52	.002
	Control	2.61 ± 1.24	2.85 ± 0.81	.227
	*p* value	.630	< .001	

### Controlling the covariate effect of scores in the pretest

In order to control and moderate the effect of scores in the pretest, an analysis of covariance (ANCOVA) was run. As reported in Table 3, the pretest scores were statistically significant covariates of perceived susceptibility (partial η2 = 0.058; *p* = 0.003), perceived barriers (partial η2 = 0.129; *p* < 0.001) and self-efficacy (partial η2 = 0.194; *p* < 0.001). However, they were not statistically significant covariates of awareness, perceived severity, benefits and behavior. As the tabulated data show, the educational intervention significantly affected awareness (partial η2 = 0.394; *p* < 0.001), all model constructs (perceived susceptibility (partial η2 = 0.592; *p* < 0.001), severity (partial η2 = 0.522; *p* < 0.001), barriers (partial η2 = 0.129; *p* < 0.001), benefits (partial η2 = 0.186; *p* < 0.001), self-efficacy (partial η2 = 0.481; *p* < 0.001)) and the behavior (partial η2 = 0.394; *p* < 0.001).

**Table 3:** Analysis of covariance to adjust the pretest scores as a variable covariate

Variables	Source	Sum of squares	df	Mean square	F-value	*p* value	Partial eta squared
Awareness	Pretes	.093	1	.093	.040	.842	.000
	Posttest	367.613	1	367.613	157.634	< .001	.517
	Error	342.814	147	2.332			
	R Squared = .518 (Adjusted R Squared = .511)						
Perceived susceptibility	Pretest	88.273	1	88.273	9.002	.003	.058
	Posttest	2091.104	1	2091.104	213.239	< .001	.592
	error	1441.541	147	9.806			
	R Squared = .603 (Adjusted R Squared = .598)						
Perceived severity	Pretest	8.403	1	8.403	1.639	.203	.011
	Posttest	824.284	1	824.284	160.754	< .001	.522
	error	753.757	147	5.128			
	R Squared = .524 (Adjusted R Squared = .518)						
Perceived benefits	Pretest	.000	1	.000	.003	.960	.000
	Posttest	4.851	1	4.851	33.639	< .001	.186
	error	21.200	147	.144			
	R Squared = .187 (Adjusted R Squared = .175)						
Perceived barriers	Pretest	397.798	1	397.798	21.760	< .001	.129
	Posttest	1243.343	1	1243.343	68.014	< .001	.316
	error	2687.269	147	18.281			
	R Squared = .373 (Adjusted R Squared = .364)						
Self-efficacy	Pretest	130.137	1	130.137	35.323	< .001	.194
	Posttest	280.681	1	280.681	76.186	< .001	.481
	error	302.570	147	3.684			
	R Squared = .461 (Adjusted R Squared = .433)						
Behavior	Pretes	.055	1	.055	.115	.735	.001
	Posttest	45.524	1	29.094	61.100	< .001	.394
	error	69.999	147	.476			
	R Squared = .403 (Adjusted R Squared = .384)						

### Predictors of adopting GWs preventive behaviors

A multivariate linear regression analysis was used to test the effect of each model construct on behavior. The dependent variable was GWs preventive behavior and awareness and other constructs were the independent variables. As it can be seen in Table 4, awareness, perceived susceptibility, severity and self-efficacy were the strong predictors of preventive behavior. The adjusted R2 value of 0.567 shows that the model explained 57% of changes in the behavior score of the intervention group.

**Table 4:** Multivariate regression analysis of the predictors of behavior in the intervention group based on the model constructs

Variables	B	95.0% confidence Interval for B		Standardized coefficients beta	t	*p* value
		Lower bound	Beta			
Awareness	.086	.035	.137	.231	3.353	.001
Perceived susceptibility	.068	.034	.103	.413	3.893	< 0.001
Perceived severity	.079	.038	.120	.315	3.779	< 0.001
Perceived Self-efficacy	.096	.045	.146	.296	3.735	< 0.001
Perceived benefits	.060	− .193	.314	.031	.471	.638
Perceived barriers	− .020	− .031	.044	.132	1.638	.104
Adjusted R Square = .567 .589 = R Square						

## Discussion

The present research investigated the effect of an educational intervention based on the HBM on adopting GWs preventive behaviors.

In the multivariate regression model, R2 was estimated at 0.57. It shows that the independent variables within the model (i.e., knowledge and HBM constructs) accounted for 57% of the variance in the dependent variable (adoption of GWs preventive behaviors).

The intervention managed to significantly affect all constructs except for the perceived benefits. As the multivariate linear regression analysis showed, awareness, perceived susceptibility and severity, and self-efficacy were the significant predictors of adopting GWs preventive behaviors.

In most studies, the educational interventions based on the HBM directly addressed HPV infection and uterine cancer screening. Therefore, we compared our findings with theirs [33–36].

As the results showed, women’s awareness both in the control and intervention groups was very low before the intervention. This was consistent with other studies showing that women’s awareness of cervical cancer screening and HPV vaccination was low before the intervention [37–39]. Still, some other studies showed divergent findings as they reported a high level of awareness of cervical cancer and Pap test administration among women [40, 41]. These contradictory findings can be explained by participants’ different demographic information, as Aweke et al. included educated women in their research, whose knowledge and awareness were certainly higher than the average population. Different purposes of research, different cultural backgrounds, and questionnaire items of different levels can partly account for contradictory findings. A systematic review attributed women’s unawareness of cervical cancer in developing countries to the lack of well-organized cancer screening programs, sociocultural barriers, and inefficient awareness-raising media [42]. Regular educational campaigns by experienced medics are highly recommended along with formal mass media to raise women’s awareness of GWs preventive behaviors.

The present findings show an increase in the awareness score of the intervention group compared to the control in the posttest. This attests to the effectiveness of the intervention. There were other works of research that confirmed these findings, as they showed an educational intervention could raise women's awareness of the Pap test administration [31, 43, 44].

Our research showed no statistically significant difference between the two groups before the intervention in terms of perceived susceptibility. This difference, however, was significant after the intervention. Similarly, a number of other studies showed that educational interventions could change women and girls’ perceived susceptibility to pap smear screening and HPV infection [45–47]. Contrary to our findings, an educational intervention was not effective in changing perceived susceptibility in another study [48]. Differences in outcomes from these studies may be due to different purposes of research, as the study by Park et al. used a short-term one-hour educational intervention aiming to increase participation in the Pap test as the researchers perceived the time of intervention inadequate for changing attitude. Also, Park et al. did not use the HBM in their study. Rather, their educational intervention was developed based on the cognition-emotion theory.

In our research, perceived susceptibility was a strong predictor of women's preventive behavior. Education can be argued to increase women's awareness of the symptoms, side effects, and preventive measures of GWs. This can increase women's susceptibility to the infection. Researchers believe to be motivated to show a particular healthy behavior, people need to be aware of the adverse effects of a disease or its effect on their awareness [49]. Resenstock (as cited in Ningrum [50]) maintained that those who perceive themselves more susceptible to a disease pay more attention to preventive and medical services [50]. In two relevant studies, perceived susceptibility showed to predict women’s adoption of healthy behavior (cancer screening and HPV vaccination) [51, 52]. We can argue that systematic and effective educational interventions made by experienced health staff can significantly affect women’s perceived susceptibility to GWs and other sexually transmitted diseases.

Moreover, our findings showed a significant increase in perceived severity scores in the intervention group in the posttest. Similarly, two other studies proved the effectiveness of educational interventions in women's perceived severity of cervical cancer screening and their intention of HPV vaccination [31, 46]. In contrast to our findings, another study reported the ineffectiveness of an educational intervention in changing women’s perceived severity of cervical cancer screening [48]. This difference can be partly explained by the different purposes of research, types of intervention and participants’ demographic features.

Our research showed that the mean perceived benefits score was not significantly different across the control and intervention groups. Also, this construct did not have any statistically significant effect on women’s preventive behaviors, which was quite expected as our intervention had no effect on this construct. On the contrary, in a number of other studies, the educational intervention showed to significantly affect the perceived benefits construct [31, 53]. We can argue that probably the benefits of adopting GWS preventive behaviors do not emerge immediately after the intervention or shortly afterwards. Thus, women are not capable of perceiving the real benefits of the target behavior. It is likely that during the intervention, the educational content did not manage to affect this construct. A short-term follow-up and the high mean score of perceived benefits before the intervention are among other reasons for this finding. It seems that designing long-term educational interventions with a longer follow-up is more realistic.

Another finding was that the mean score of perceived barriers was decreased in the intervention group compared to the control in the post-test. Similarly, in some other studies, the mean score of perceived barriers was lowered after the educational intervention [31, 48]. Hyacinth et al. reported that perceived barriers was the strongest predictor of administering the Pap test. This can show that removing barriers to the administration of the Pap test can increase the rate of administering the test [54]. Contrary to the present findings, in another study, the educational intervention had no effect on the mean score of perceived barriers [46]. In our research, though the mean score of perceived barriers was reduced in the intervention group, it had no effect on women's healthy behavior. The absence of any increase in the perceived benefits score can be possibly another explanation for this. Probably, when women do not perceive the benefits of behavior, the barriers to adopting the healthy behavior are highlighted, and, thus, women do not make any attempts to lower the barriers.

Our findings showed an increase in the mean score of self-efficacy in the intervention group in the posttest. Similar to the present findings, some other studies showed that high self-efficacy managed to increase the intention of HPV vaccination and administering the Pap test [48, 53]. Exploration of the association between self-efficacy and healthy behavior showed that the former strongly affected the latter. It is less likely that someone with a lower self-efficacy changes an already established behavior or show a new healthy behavior [55]. The increased self-efficacy and its predictive effect on the adoption of GWs preventive behaviors were quite expected in this research. The participants tried to use a systematic and theory-based educational intervention specifically beside the other constructs as self-efficacy plays a significant role in the adoption of a healthy behavior such as HPV vaccination and administration of the Pap test [55, 56].

Our research showed an increase in the mean score of women's self-reported behaviour in the intervention group compared to the control. This proves the effectiveness of the educational intervention based on the HBM in adopting GWs preventive behavior. A number of other studies also confirmed this finding that an educational intervention can positively affect women’s healthy behavior [43, 57]. Arguably, raising awareness through affecting other HBM variables can positively affect women’s adoption of healthy behavior.

## Limitations, strengths and future research

The small sample size in the present research limits the generalization of results to women in other provinces and target populations, including men. To increase the generalizability of results, future research is required with a larger sample with male and female participants both in different geographical areas with different sociocultural features. GWs preventive behavior needs to be explored more thoroughly. The absence of any similar research limits the comparability of results and decision-making in the field. The short-term follow-up of women is another limitation of this research. Thus, it is recommended that the participants be followed for at least a year to check the consistency of the behavior. Besides these limitations, there are certain strengths too. For one, The HBM is a systematic model to explain a healthy preventive behavior. This model encompasses key concepts in the intervention and elaborates on them [46]. Our research can act as a basis of comparing other studies and models in near future. It also provides key information for policy-makers about the significance of health education in the adoption of preventive health behavior including GWs.

## Conclusion

Our results showed that women’s awareness of different aspects of GWs was low. This low awareness was a warning for the promotion of healthy behaviors among women. That is because a low awareness of women is followed by a lower perceived severity of the GWs. These all make women unaware of the benefits of the above-mentioned healthy behavior. When they fail to perceive the benefits, they perceive more barriers and make no attempts to remove them. All these factors together weaken women’s capability of and determination in showing the desired healthy behavior. Our findings showed that an educational intervention based on the HBM can increase the acceptability of GWs preventive behaviors among women. The education should be provided by experienced experts regularly at short intervals in all healthcare centers. We, in particular, hope to implement systematic educational programs in the light of health education and promotion models to promote GWs preventive behaviors. Health education specialists and local media can be actively involved in the success of these educational programs.

## Supplementary Information

**Additional file 1**. The Educational Intervention Content.

## Data Availability

The datasets used and analysed during the current study are available from the corresponding author on reasonable request.

## Abbreviations

GWs: Genital warts
HBM: Health belief model
