# Supplementary material for: The effectiveness of a model-based health education program on genital warts preventive behaviors: a quasi-experimental study
Source: Infect Agent Cancer. 2021 Dec 11;16:68. doi: 10.1186/s13027-021-00408-w (PMC8666010; doi:10.1186/s13027-021-00408-w)
Supplement: Supplementary file 1 — Additional file 1. The Educational Intervention Content. [file 13027_2021_408_MOESM1_ESM.docx]

| **The Educational Intervention Content** | | |
| --- | --- | --- |
| **Timing** | **Lesson** | **Activities** |
| 1^st^ week: 60 minutes | Familiarity with the purpose of educational intervention, expectations and the process of educational intervention, gaining participants’ trust | In this session, the teacher introduces the purpose and significance of research. She elaborates on the teaching methods and syllabus. She uses a predetermined checklist to review the target teaching methods and content according to the participants’ feedback. |
| 2^nd^ week: 60 minutes | Familiarity with HPV infection and all symptoms of GWs | In this session, the participating women get to know about GWs through lectures, brainstorming, active and cooperative discussions, and images shown through an OHP. The evaluation checklist is used at the end of the session to find the defects and make up for them. |
| 3^rd^ week: 40 minutes | Familiarity with the ways of transmitting GWs | In this session, women come to know the ways of transmitting GWs through lectures, active discussions and brainstorming. |
| 4^th^ week: 60 minutes | Familiarity with the underlying factors of GWs infection | In this session, women learn about the risk factors of GWs through lectures, active discussions and brainstorming. At the end of class, an evaluation checklist is used to find the defects and make up for them. |
| 5^th^ week: 40 minutes | To continue with introducing the factors underlying GWs | In this session, women learn about the risk factors of GWs through lectures, active discussions and brainstorming. |
| 6^th^ week: 60 minutes | Familiarity with how to prevent the GWs infection, significance of the Pap test, authorized places to hold the Pap test | In this session, women learn about how to prevent the occurrence of GWs infection through lectures, active discussion, and brainstorming. |
| 7^th^ week: 40 minutes | Significance of preventing GWs | In this session, women learn about the significance of preventing the occurrence of GWs through lectures, active discussion, and brainstorming. |
| 8^th^ week: 40 minutes | Short-term and long-term effects of GWs | In this session, women come to know about the adverse effects of GWs through lectures, active discussion, and brainstorming. |
| 9^th^ week: 40 minutes | To continue with the elaboration of the long-term and short-term effects of GWs | In this session, women listen to doctors’ speech on national media and the facts and figures about the adverse effects of GWs. |
| 10^th^ week: 40 minutes | Advantages of adopting GWs preventive behaviors | In this session, women learn about the advantages of adopting GWs preventive behaviors through active discussion and brainstorm |
| 11^th^ week: 40 minutes | To teach useful strategies to remove barriers to the adoption of GWs preventive behaviors | In this session, active cooperative discussions and brainstorming are used to enlist the perceived barriers to the adoption of healthy behaviors. Then a brainstorming is used to find ways to remove these barriers. |
| 12^th^ week: 40 minutes | To motivate women to adopt GWs preventive behaviors | In this session, the physical and social benefits of the absence of GWs are pinpointed by the teacher to motivate them. Attention is also drawn to the value of human body and the need to take care of it. |
| 13^th^ week: 40 minutes | To increase self-efficacy | The teacher draws attention to the existing benefits and barriers as well as the effective strategies to increase women’s self-efficacy. |
| 14^th^ week: 40 minutes | To continue with increasing self-efficacy | In this session, the role model and different types of strategies are used to increase women’s self-efficacy. |
| 15^th^ week: 40 minutes | To review all educational content taught by the gynecologist | In this session, the gynecologist reviews the previous content on the significance of adhering to genital health recommendations. She provides evidence for the GWs leading to cervical cancer. She discusses the severity of the infectious disease. |
